# Supplementary material for: Macrophage-P2X4 receptors pathway is essential to persistent inflammatory muscle hyperalgesia onset, and is prevented by physical exercise
Source: PLoS One. 2025 Feb 11;20(2):e0318107. doi: 10.1371/journal.pone.0318107 (PMC11813081; doi:10.1371/journal.pone.0318107)
Supplement: S1 Fig — Representative image of total protein loading stained by Ponceau S dye in the membranes containing skeletal muscle samples of Day 0 (A, B, E and F) and Day 1 (C, D, G and H) after Saline (Sal) or Carrageenan (Cg) injections. B, D, F and G represent the membranes probed with anti-p-p38 antibody. A, C, E and G represent membranes that were probed with anti-p38 antibody. A–D include non-exercise animals with and without the 5-BDBD strategy. E–H include exercised animals with and without GW9662 strategy. The number marking indicates the height of the marker in kilodaltons (kDa). Numbers on the lanes indicate the biological replicates in the gel. (PDF) [file pone.0318107.s001.pdf]

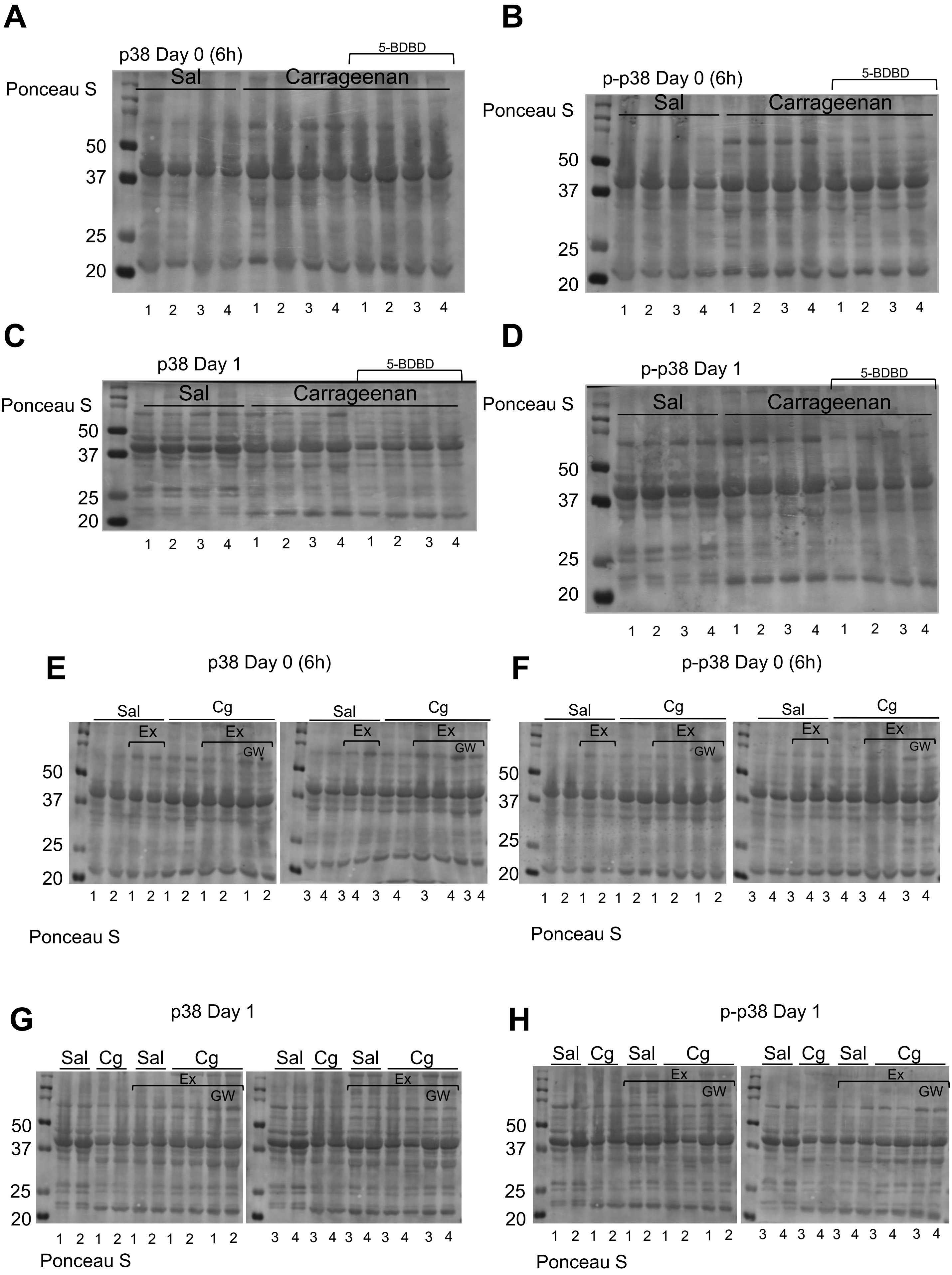
A
p38 Day 0 (6h)
5-BDBD
Ponceau S
Sal
Carrageenan
50
37
25
20
1 2 3 4 1 2 3 4 1 2 3 4
B
p-p38 Day 0 (6h)
5-BDBD
Ponceau S
Sal
Carrageenan
50
37
25
20
1 2 3 4 1 2 3 4 1 2 3 4
C
p38 Day 1
5-BDBD
Ponceau S
Sal
Carrageenan
50
37
25
20
1 2 3 4 1 2 3 4 1 2 3 4
D
p-p38 Day 1
5-BDBD
Ponceau S
Sal
Carrageenan
50
37
25
20
1 2 3 4 1 2 3 4 1 2 3 4
E
p38 Day 0 (6h)
Sal
Cg
Ex
Ex
GW
Sal
Cg
Ex
Ex
GW
50
37
25
20
1 2 1 2 1 2 1 2 1 2
3 4 3 4 3 4 3 4 3 4
Ponceau S
F
p-p38 Day 0 (6h)
Sal
Cg
Ex
Ex
GW
Sal
Cg
Ex
Ex
GW
50
37
25
20
1 2 1 2 1 2 1 2 1 2
3 4 3 4 3 4 3 4 3 4
Ponceau S
G
p38 Day 1
Sal Cg Sal Cg
Ex
GW
Sal Cg Sal Cg
Ex
GW
50
37
25
20
1 2 1 2 1 2 1 2 1 2
3 4 3 4 3 4 3 4 3 4
Ponceau S
H
p-p38 Day 1
Sal Cg Sal Cg
Ex
GW
Sal Cg Sal Cg
Ex
GW
50
37
25
20
1 2 1 2 1 2 1 2 1 2
3 4 3 4 3 4 3 4 3 4
Ponceau S

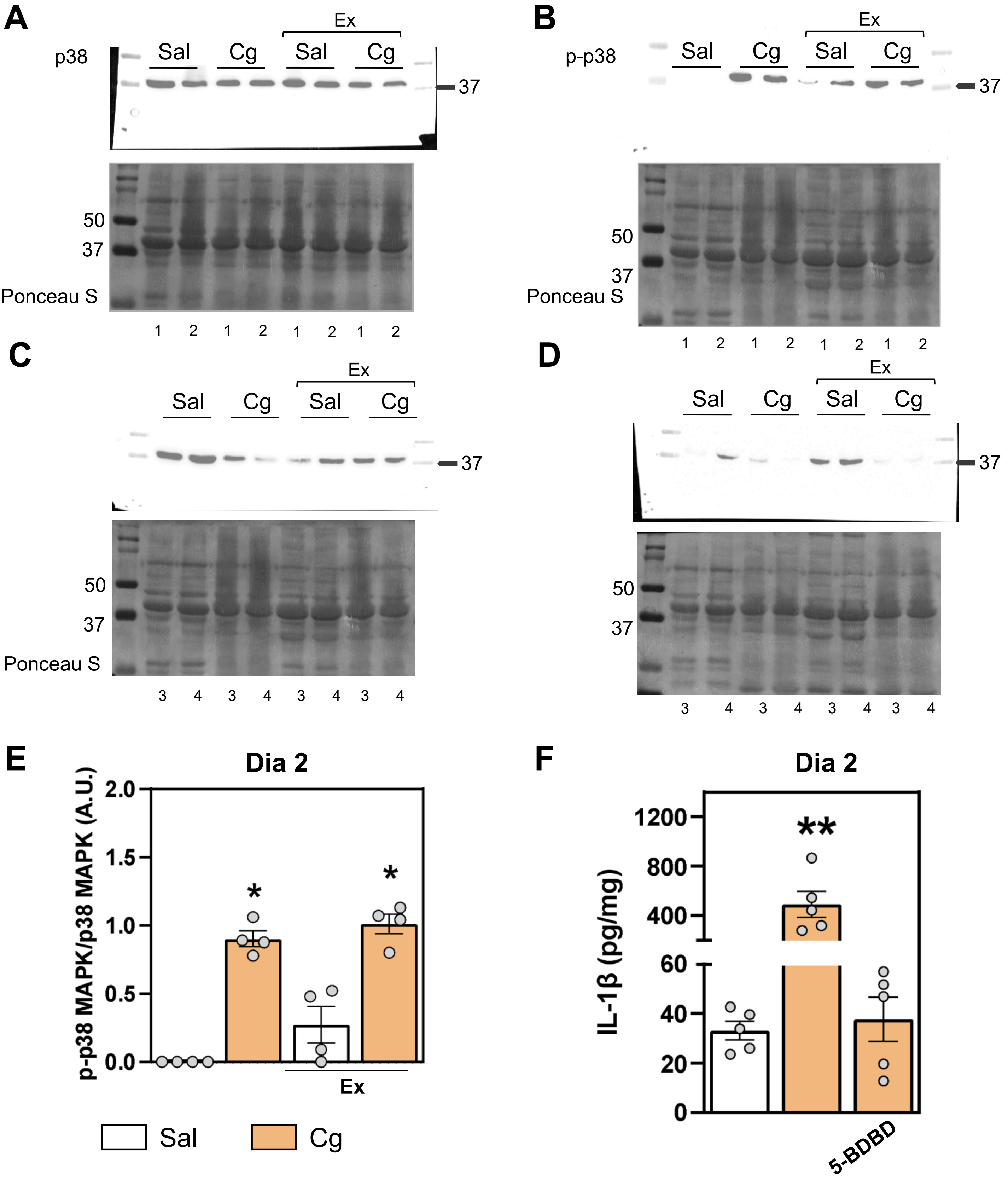
A
p38
Sal
Cg
Ex
Sal
Cg
37
50
37
Ponceau S
1 2 1 2 1 2 1 2
B
p-p38
Sal
Cg
Ex
Sal
Cg
37
50
37
Ponceau S
1 2 1 2 1 2 1 2
C
Sal
Cg
Ex
Sal
Cg
37
50
37
Ponceau S
3 4 3 4 3 4 3 4
D
Sal
Cg
Ex
Sal
Cg
37
50
37
3 4 3 4 3 4 3 4
E
Dia 2
p-p38 MAPK/p38 MAPK (A.U.)
2.0
1.5
1.0
0.5
0.0
*
*
Ex
Sal
Cg
F
Dia 2
IL-1β (pg/mg)
1200
800
400
60
40
20
0
**
5-BDBD

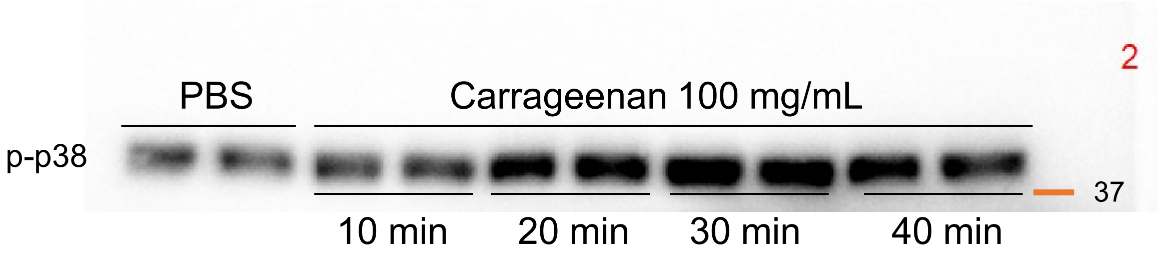
2
PBS
Carrageenan 100 mg/mL
p-p38
37
10 min
20 min
30 min
40 min

Raw images for Figure 4 panel B

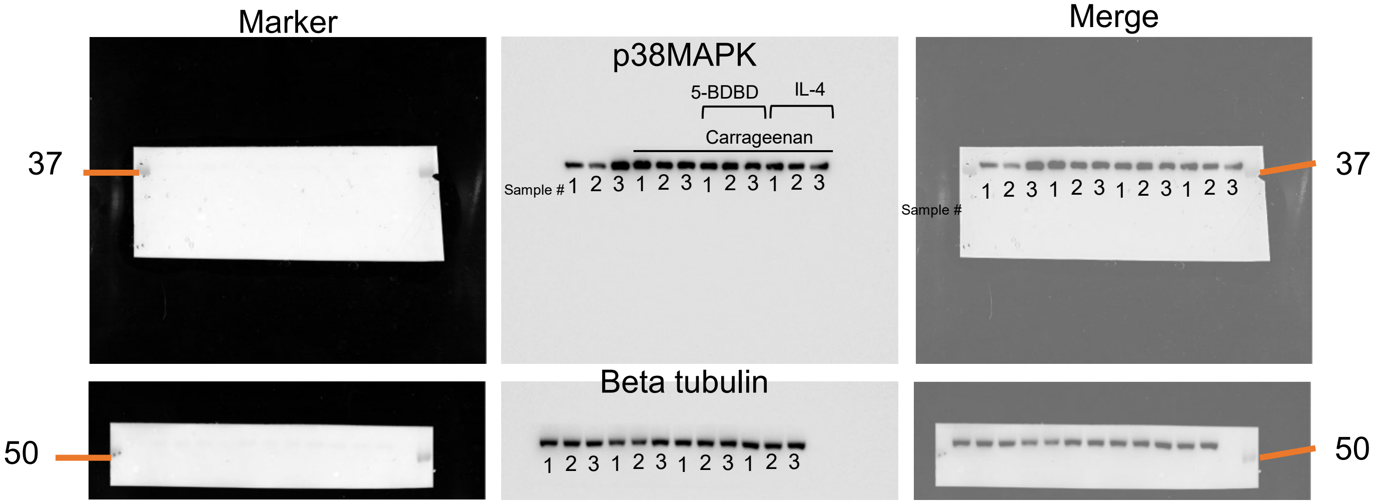

Raw images for Figure 4 panel B

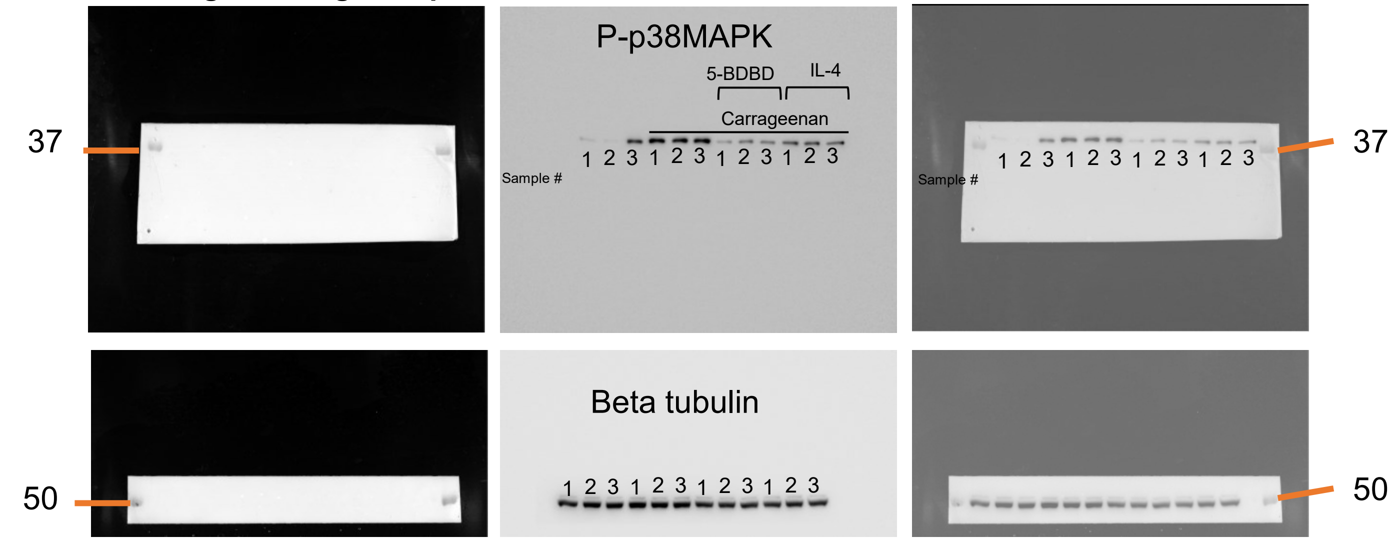

A p38 Day 0 (6h)

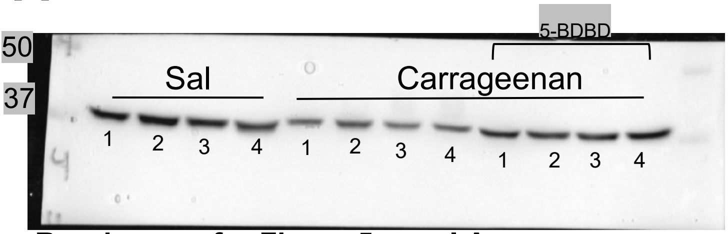

Raw images for Figure 5 panel A

B p-p38 Day 0 (6h)

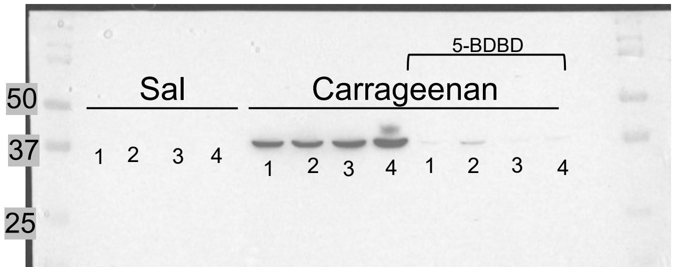

Raw images for Figure 5 panel A

C p38 Day 1

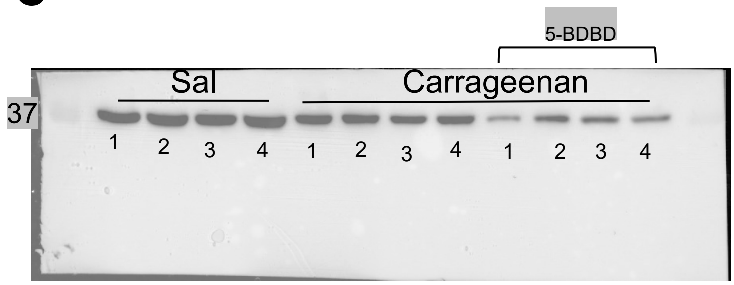

Raw images for Figure 5 panel C

D p-p38 Day 1

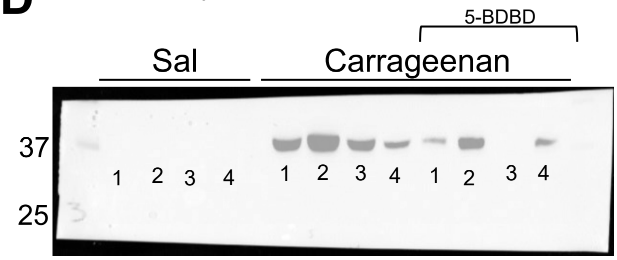

E p38 Day 0 (6h)

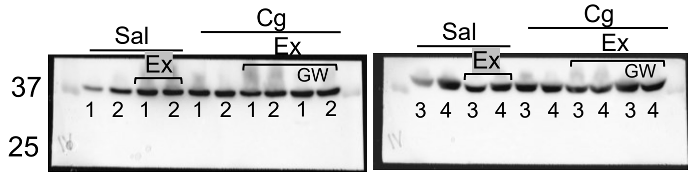

Raw images for Figure 6 panel A and graph in B

F p-p38 Day 0 (6h)

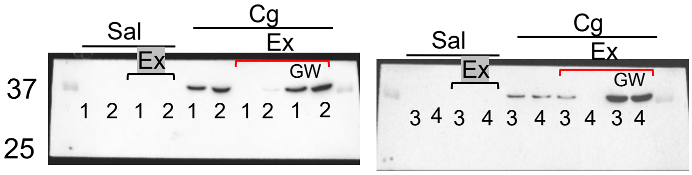

Raw images for Figure 6 panel A and graph in B

G p38 Day 1

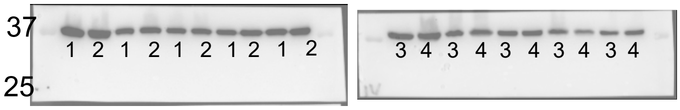

Raw images for Figure 6 panel C and graph in D

H p-p38 Day 1

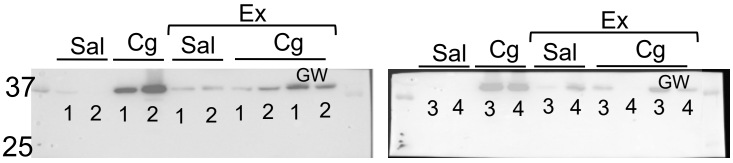

Raw images for Figure 6 panel C and graph in D
