# Supplementary figures and images for: Macrophage-P2X4 receptors pathway is essential to persistent inflammatory muscle hyperalgesia onset, and is prevented by physical exercise
Source: PLoS One. 2025 Feb 11;20(2):e0318107. doi: 10.1371/journal.pone.0318107 (PMC11813081; doi:10.1371/journal.pone.0318107)

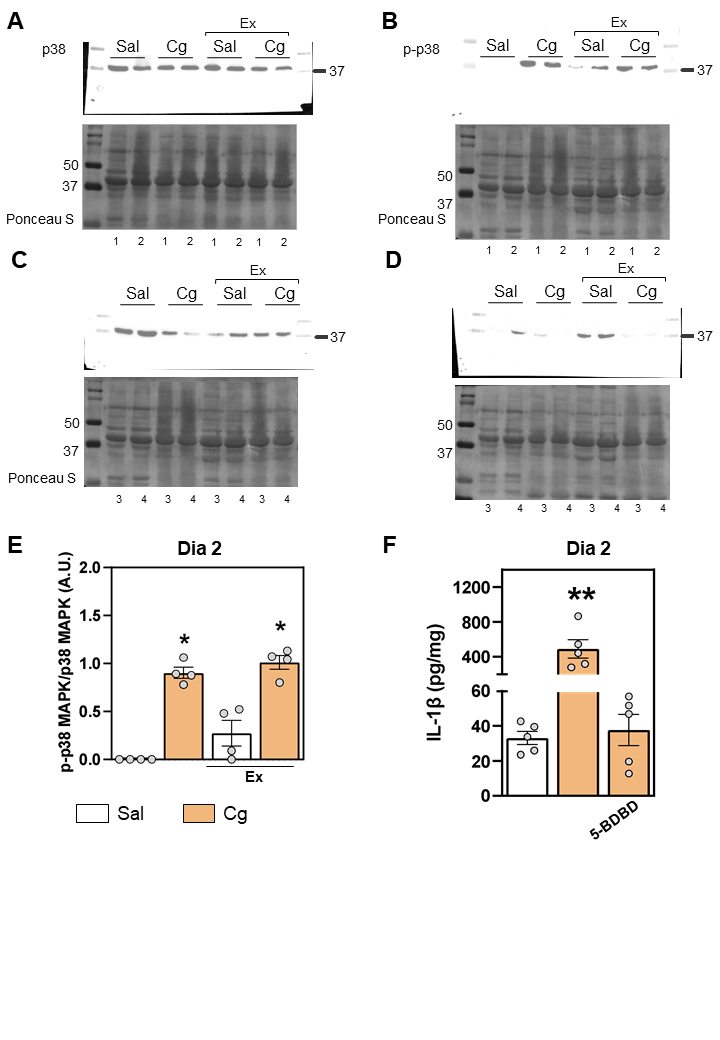

Supplement: S2 Fig — A–D) Representative image of western blotting membranes demonstrating expression of p38 MAPK, p-p38 MAPK proteins and ponceau staining on day 2, in Control and Exercise (Ex) groups injected with saline (Sal) or carrageenan (Cg). Total protein staining for each membrane are presented by the Ponceau S images. The number marking indicates the height of the marker in kilodaltons (kDa). The numbers on the lanes indicates the biological replicates. Original unedited membranes. Full membranes were imaged through chemiluminescent detection for 10–20 seconds. E) Expression of p-p38 MAPK/p38 MAPK (arbitrary units [A.U.]) on day 2 (two-way ANOVA, Tukey post hoc). “*” indicates difference with Control Sal and Exercise Sal groups, and “+” difference with Exercise Cg group. N = 4 for each group. F) Expression of the cytokine IL-1β in picograms/milligrams of total protein (pg/mg) in the muscle tissue at day 2 after the injection in controls saline (Sal) and Carrageenan (Cg), and in a group that received 5-BDBD previous to Cg. Symbol “**” indicates difference with Sal and Cg groups (one-way ANOVA, Tukey post hoc). (TIF) [file pone.0318107.s002.TIF]

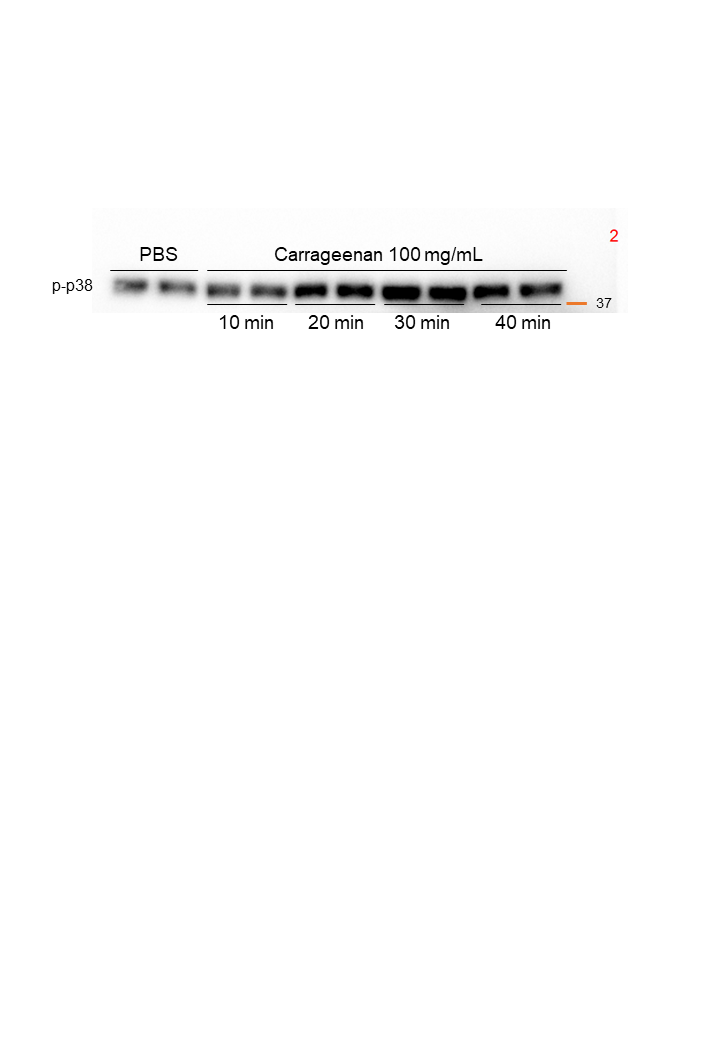

Supplement: S3 Fig — Representative image of western blotting membrane demonstrating expression of p-p38 MAPK over time after carrageenan stimulation, shows increased p-p38 MAPK expression at 30 min after inflammatory stimulus. (TIF) [file pone.0318107.s003.TIF]

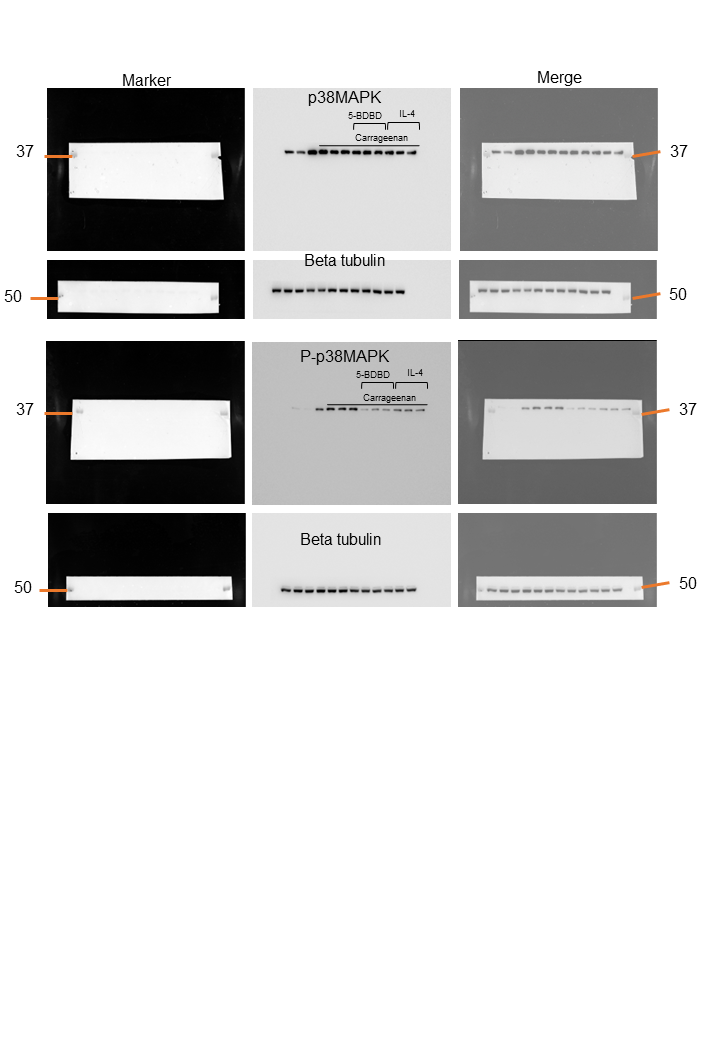

Supplement: S4 Fig — Fig 4. Western blotting membranes demonstrating the expression of p38 MAPK, p-p38 MAPK and endogenous beta tubulin proteins, in RAW 264.7 treated with PBS, carrageenan (Cg), 5-BDBD + Cg or IL-4 + Cg. The number marking indicates the height of the marker in kilodaltons (kDa). Numbers on the lanes indicate biological replicates. Original unaltered images. Full membranes were imaged through chemiluminescent detection for 10–20 seconds. (TIF) [file pone.0318107.s004.TIF]

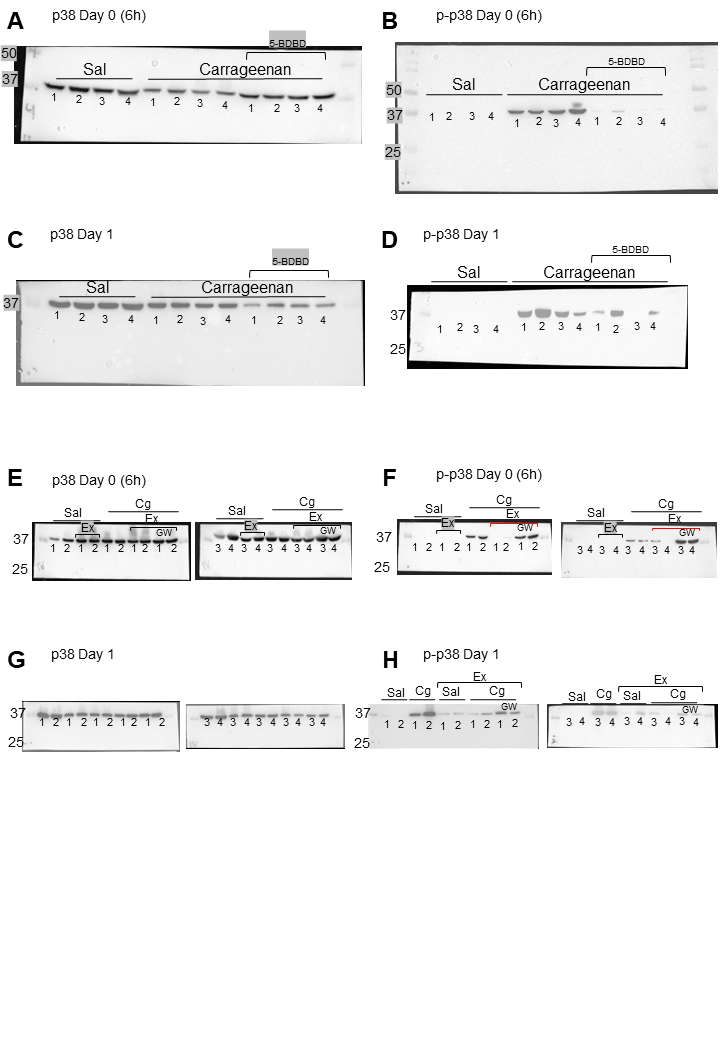

Supplement: S5 Fig — Figs 5 and 6. A–D) Western blotting membranes demonstrating the expression of p38 MAPK, p-p38 MAPK proteins at days 0 (6h) and 1, in gastrocnemius muscle of mice treated with Saline (Sal), carrageenan (Cg) or 5-BDBD + Cg. E–H) Western blotting membranes demonstrating the expression of p38 MAPK, p-p38 MAPK proteins at days 0 (6h) and 1, in gastrocnemius muscle of sedentary and exercised (Ex) mice injected with Sal, Cg or GW9662 (GW) + Cg. The number marking on the side indicates the height of the marker in kilodaltons (kDa). Numbers on the lanes indicate biological replicates. Original unaltered images. Full membranes were imaged through chemiluminescent detection for 10–20 seconds. (TIF) [file pone.0318107.s005.TIF]
